# The urgency in proposing the optimal obesity cutoff value in Indonesian population: A narrative review

**DOI:** 10.1097/MD.0000000000032256

**Published:** 2022-12-09

**Authors:** Gaga Irawan Nugraha, Dicky L. Tahapary, Rachmad Wishnu Hidayat, Nurul Ratna M. Manikam, Mas Rizky A.A. Syamsunarno, Farid Kurniawan, Errawan R. Wiradisuria, Dwi Yuniati Daulay, Dante Saksono Harbuwono, Sidartawan Soegondo

**Affiliations:** a Indonesian Society for the Study of Obesity; b Department of Biomedical Science, Faculty of Medicine, Universitas Padjadjaran, West Java, Indonesia; c Division of Endocrinology, Metabolism, and Diabetes, Department of Internal Medicine, Dr. Cipto Mangunkusumo National General Hospital, Faculty of Medicine Universitas Indonesia, Jakarta, Indonesia; d Division Sports Medicine Department Community Medicine, Faculty of Medicine Universitas Indonesia, Jakarta, Indonesia; e Department of Nutrition, Faculty of Medicine, University of Indonesia, Dr Cipto Mangunkusumo Hospital, Jakarta, Indonesia; f Division of Digestive Surgery, Mayapada Hospital, South Jakarta, Indonesia; g Indonesian Metabolic Bariatric Society.

**Keywords:** body mass index, cutoff, Indonesia, obesity, staging

## Abstract

In developing nations such as Indonesia, obesity and central obesity have emerged as major public health issues. Many studies have revealed that morbidity and death from obesity-related diseases are already significant in some “Asian” communities at low body mass index (BMI) levels. A recent study showed that the obesity prevalence in Indonesia is underestimated when using the current BMI cutoff (obese ≥ 27.0). Indonesia faced an increase in obesity-related chronic diseases despite having a lower obesity prevalence than developed countries, which may be explained by the underestimation of obesity levels in Indonesia. This creates a huge global health problem, as well as an economic burden. Another recent study on the Indonesian population depicted the new proposed cutoff of waist circumference (WC), which is lower than the World Health Organization (WHO) standard for detecting the early detection of type 2 diabetes mellitus (T2DM), one of the comorbidities and a strong correlation with obesity. An analysis of 58 studies in 2021 that included Indonesian adult subjects revealed enormous differences and ambiguities in defining obesity cutoffs values among Indonesian researchers. Additionally, we advocate adding the Edmonton Obesity Staging System (EOSS) staging to the anthropometric classification for a better clinical evaluation of obesity. Considering the urgency of obesity determination in Indonesia for clinical application and study purposes, this review highlights the need to revise the optimal cutoff value for obesity to warrant early prevention and control of diabetes complications.

## 1. Introduction

Obesity is characterized by the excessive accumulation of body fat tissue and has become a worldwide health problem. In Indonesia, the prevalence of obesity in the adult population increased from 10.3% (2007) to 23.1% (2018) based on the results of the Basic National Health Survey.^[1–3]^ This increase is believed to be due to a sedentary lifestyle and lack of awareness of a healthy diet.^[4–6]^ On the other hand, the dramatic increase also raises serious concerns about the potential increase in of obesity-related chronic diseases, such as type 2 diabetes,^[7,8]^ cardiovascular diseases^[9]^ and cancer,^[10–12]^ creating a huge global health problem. This could be due to poor management or underestimation of obesity levels itself by only considering the body mass index (BMI).^[13]^ An appropriate management of obesity should implicate lifestyle modification (physical activities, diet, and behavior modification), pharmacotherapy and metabolic surgery (bariatric procedures).

The analysis of obesity studies in Indonesia that included 56 articles showed us that there are enormous differences in defining obesity cutoff values. This interim analysis bolsters the concept ambiguities still exist among Indonesian researchers. Furthermore, previous studies have shown that the Asian population has an increased risk of adiposity-related morbidity and mortality with lower BMIs and smaller waist circumference (WC) compared to Caucasians.^[14]^ A recent study showed that the obesity prevalence in Indonesia is underestimated when using BMI (obese ≥ 27.0), since it misclassified 40% of obese people as normal or overweight.^[13]^ Despite having a lower obesity prevalence than developed countries, increased chronic disease may be explained by Indonesia’s obesity levels being underestimated.^[1,3,15,16]^ Another recent study on the Indonesian population depicted the new proposed cutoff of WC cutoff (76 cm for men and 80 cm for women), which is lower than the World Health Organization (WHO) standard.^[7,17]^ However, this newly proposed WC cutoff is used for the early detection of type 2 diabetes mellitus (T2DM), where we know that T2DM is only one of the obesity comorbidities. Thus, the present study was designed to promote the necessity of revising the cutoff value for obesity among Indonesian populations. Moreover, there are currently no standard clinical guidelines for diagnosing obesity in Indonesia.

Hitherto, there is no agreement among the government, physicians, and researchers on the adult cutoff value that acts as a national representative. Changing the BMI cutoff points that define obesity to account for this effect produces a higher prevalence estimate and possibly a better assessment of the health risks associated with obesity in Indonesia.

## 2. Obesity study in Indonesia

**Figure 1.**
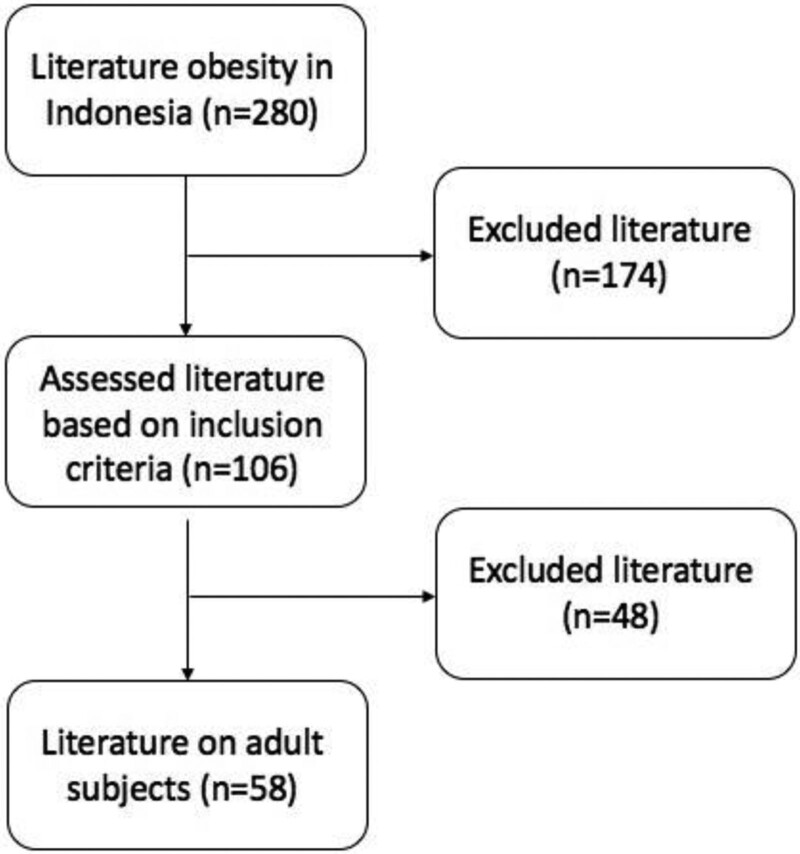
Flow charts of literature selection.

We analyzed the literature trend of obesity from Indonesian publishers indexing in google scholar using keywords of “Obesitas” (Obesity) AND “Indonesia” in 2021. We found 280 articles, from these, we excluded studies that did not measure body weight and height ratio, case reports, review and meta-analysis studies, in vivo and in vitro studies, repository articles, inaccessible full articles and articles that used secondary data from national health data. A total of 106 studies fulfilled the inclusion criteria. However, only 58 articles have evaluated the adult subjects (Fig. 1). We further analyzed the BMI as the obesity cutoff point in adults. Forty-four percent (26/58) of the obesity studies in adults used a cutoff of ≥25 kg/m^2^ and Asia Pacific WHO standard for obesity.^[18]^ Additionally, 1, 6, and 7 studies used cutoffs of ≥23, ≥27, and ≥30 kg/m^2^, respectively. In contrast, 18 studies did not mention BMI cutoff points. This interim analysis of obesity studies in Indonesia showed us that there are enormous differences and ambiguities in defining obesity cutoffs values among Indonesian researchers. Considering the urgency of obesity determination in Indonesia for clinical application and study purposes, it is pivotal to have a standard cutoff point and the same perspective in BMI measurement.

## 3. Obesity is a disease

### 3.1. Premise

Obesity was officially declared a disease in June 2013 by the American Medical Association. Previously, obesity became controversial since it did not meet the definition of the disease. However, some studies have shown that obesity increases the risk of multiple diseases such as cardiovascular diseases, hypertension, stroke, T2DM, degenerative arthritis, colon, and breast cancer.^[19]^ Moreover, obesity is predicted to cause approximately 112,000 to 365,000 deaths annually.^[20]^ The Council of the Obesity Society believes that declaring obesity as a disease would benefit society by seeking more information on preventing and treating obesity. It also encourages health providers to treat obesity by reducing the stigma and discrimination faced by people with obesity.^[21]^

In 2017, The American Association of Clinical Endocrinologists (AACE) and American College of Endocrinology (ACE) created a new term “adiposity-based chronic disease” (ABCD) that stresses on complications-centric approach as a new diagnostic term for obesity that recognizes it as a chronic disease and to avoid the stigma and ambiguity associated with the term’s numerous connotations.^[22]^ Later in 2018, AACE also created the term “dysglycemia-based chronic disease” (DBCD), a multimorbidity care model with 4 stages that can be addressed in a preventive care paradigm to lower the risk of T2DM, cardiometabolic risk, and cardiovascular events.^[23]^ The 4 stages include, stage 1 “insulin resistance,” stage 2 “prediabetes,” stage 3 “type 2 diabetes,” and stage 4 “vascular complications.” At the level of insulin resistance, the natural history of ABCD connects with that of DBCD, in which metabolic syndrome is usually caused by insulin resistance in the presence of being overweight or obese. Furthermore, in 2020 a new model called “cardiometabolic-based chronic disease” (CMBCD) was presented based on of promoting cardiometabolic health that focuses on 3 primary drivers (environment, genetics and behavior) and 2 metabolic drivers (adiposity and dysglycemia) to 3 cardiovascular endpoints (heart failure, coronary heart disease and atrial fibrillation). These 2 metabolic drivers, along with the primary drivers, lead to the progression of CMBCD since ABCD and DBCD cross at the level of insulin resistance to aggravate CMBCD.^[24]^

### 3.2. Pathogenesis

The pathogenesis of obesity is a complex process. It involves a multitude of factors such as calorie consumption, exercise, health-care availability, genetics, socio-economic status and environmental factors.^[25]^ A study of 3000 people with 3-year follow-up delineated that those who consume more frequent fast food have a larger waist and higher BMI than those who consume minimal fast food.^[26]^ A child with one or both obese parents will have a 3- and 10-time risk of being obese in the future.^[27]^ Together with the hereditary factor, the technological progress nowadays has deteriorated our young generations. Children tend to spend more time on video games and mobile phones, thus reducing the time spent on exercising. Next, is the correlation of genetic role with obesity, the cause can largely be categorized as monogenic origin resulting from a single gene mutation, polygenic origin due to the collective contribution of many genes, and severe syndrome obesity that is caused by neurodevelopmental abnormalities and organ malformation. Intriguingly, variations in human obesity resulting from genetic factors accounted for 40% to 70%.^[28]^ The imbalance of gut microbiota (dysbiosis) is also thought to play a role in developing obesity and its metabolic complications. A study in a rodent model showed an overt increase in the weight of lean germ-free mice after transplanted with feces from obese mice. On the contrary, the obese mice were observed to show a trend toward weight loss after being transplanted with feces from lean mice.^[29]^ In addition, the gut microbiome can produce short-chain fatty acids (SCFAs), which can regulate intestine hormones such as peptide YY (PYY) and glucagon-like peptides-1 (GLP-1), GLP-2, and the emission of gastric inhibitory peptides by K cells.^[30]^ In obese patients, glucose signaling pathways and production of SCFAs are downregulated.

### 3.3. Comorbidity and complications

Obese patients are more likely to develop comorbidities and complications such as dyslipidemia, T2DM, hypertension, coronary heart disease, stroke, gallbladder disease, respiratory issues, sleep apnea, osteoarthritis, and malignancies.^[1,31]^ Compared to normal weight, adults with a BMI ≥ 40 kg/m^2^ had 7, 6, 4, 3, and 2 times higher risk developing diabetes, hypertension, arthritis, poor or fair health, asthma, and hyperlipidemia, respectively.^[32]^ A study (N = 300) comparing siblings with and without hypertension has found genetic link between obesity and hypertension. Siblings with hypertension were more obese and had more centrally distributed body fat than their non-hypertensive siblings.^[33]^ In a cross-sectional study based on the 2013 National Basic Health Survey, hypertension (61%) and hyperglycemia (51%) were the 2 most prominent components found in people with metabolic syndrome.^[16]^ A study in Indonesia that analyzed the relationship between BMI and DM incidence in 22,647 subjects, showed that obese people had a higher chance of acquiring diabetes than those who were underweight (OR:3.15; 95% CI = 2.05–4.82).^[31]^ However, this study only classified BMI into 3 categories: underweight (BMI < 18.5), normal (BMI 18.5–25) and obese (BMI ≥ 25). Moreover, a systematic review also outlined obesity as a risk factor for advanced medical care requirements (OR:7.36; 1.63–33.14; *P* = .021) and a higher mortality rate in COVID-19 patients.^[34]^

In addition to predicting the risk of occurrence of obesity-related disease, a meta-analysis of the population of the Asian Pacific region showed that when compared to BMI and hip circumference (HC), WC and weight-hip ratio (WHR) are better predictors of coronary risk, which was in line with the results from the North American and European populations. Meanwhile, BMI, WHR, and WC are more robust predictors of ischemic heart disease than HC.^[35]^ A recent study found that 90% of obese patients with obesity have cardiometabolic risk based on body fat% (men ≥ 25.8%, women ≥ 37.1%), which is in line with BMI. Meanwhile, only 40% to 70% had cardiometabolic risk when WC, WHR and weight-to-height ratio (WHTR) were applied.^[36]^ A 16-year follow-up study of the Chinese population (BMI ≥ 24 kg/m^2^ for overweight and ≥ 28 kg/m^2^ for obesity, respectively) showed that when compared to a low stability group, a group that remained highly stable in fat mass to fat-free mass ratio (F2FFMR) had a significant association with diabetes and hypertension.^[37]^

## 4. Diagnosis and clinical evaluation

### 4.1. Cutoff

Various health organizations have proposed different standards for adult BMI in adults. The WHO defines overweight and obesity as BMI ≥ 25 kg/m^2^ and ≥ 30 kg/m^2^, respectively. The degree of obesity is then further subcategorized into class I (30–34.9 kg/m^2^), class II (35–39.9 kg/m^2^) and class III (≥40 kg/m^2^).^[38]^ Furthermore, in 2002, WHO proposed a lower BMI cutoff for Asians, defining overweight and obese as BMI of ≥23 kg/m^2^ and ≥27.5 kg/m^2^, respectively.^[14]^ An earlier study^[39]^ was assumed as precedent work by the Indonesian Ministry of Health, corroborating the decision to propose a new cutoff value for overweight and obese as ≥25 and ≥27 kg/m^2^, respectively.^[40]^ Hitherto, this cutoff is still adopted by the Indonesian Ministry of health. Meanwhile, the Asian-Pacific recommends lower cutoffs for overweight (≥23 kg/m^2^) and obese (≥25 kg/m^2^).^[18]^ A substantial body of research has supported these cutoffs. Data from Indonesia showed that obesity prevalence was severely underestimated, in which 40% of the obese individuals (BMI ≥ 27 kg/m^2^) were categorized as normal or overweight. Hence, the study suggested using WC in clinical settings to identify individuals with high visceral adipose tissue that might lead to an increase in chronic diseases.^[13]^ Another recent study among the Indonesian population that included 24,660 adult participants also delineated that when compared to BMI, WC (men = 76, women = 80) would be a better predictor for measuring central obesity to identify T2DM.^[7]^

Knowing that Indonesia is a multi-ethnic country, the incidence of obesity using recommended cutoffs of obese as ≥25 kg/m^2^, may provide early detection of obesity and chronic obesity-related diseases, thus preventing the occurrence of obesity-related complications. Studies among the Chinese population living in Hongkong,^[41]^ Singapore^[42]^ and Indian Asians living in Mauritius indicated a cutoff point of BMI > 23 kg/m^2^ increased the risk of morbidity and significantly increased the risk of T2DM and hypertension. A large population study in Malaysia (n = 32703) also suggested that a BMI cutoff of 23 kg/m^2^ for men and 24 kg/m^2^ for women is the optimal cutoff value for predicting risk factors for diabetes, hypertension and hypercholesterolemia.^[43]^

### 4.2. Staging

BMI and WC are the common classification measures for obesity. However, individuals with the same BMI can have very different health conditions depending on several factors other than body weight.^[44,45]^ As a corollary, a one-size-fits-all approach to obesity treatment is insufficient, and a more personalized and targeted approach is needed to improve the health of those with the most severe obesity conditions. Another limitation of the current anthropometric metrics of obesity is that they have limited evidence and do not account for the presence of comorbid conditions or disease risk, which, according to current guidelines, should be taken into account when making treatment decisions.

The Edmonton Obesity Staging System (EOSS) is a classification system that includes metabolic, physical, and psychological factors for clinical evaluation, to provide the best obesity intervention options. It categorizes obesity into 5 stages (stage 0–4), stage 0 indicates no obesity-related risk factors or any health impairments; and stage 4 indicates severe disabilities from obesity-related chronic diseases.^[46]^ The risk of chronic diseases such as T2DM, coronary heart disease, and stroke rises exponentially with BMI units,^[47]^ the WHO recognizes that these reported dangers occur at lower BMI cutoff lines for overweight and obesity in certain Asian communities.^[14]^ We suggest adding EOSS to the anthropometric classification in Indonesia since EOSS could be a useful method in stratifying the presence and severity of weight-related health concerns and has been deemed to be a better predictor of mortality than BMI.^[48,49]^

## 5. Conclusion

In conclusion, this review suggests that revising the BMI cutoff value of ≥25 kg/m^2^ may be more appropriate for defining obesity in the Indonesian adult population. We also advocate adding EOSS staging to the anthropometric classification for a better clinical evaluation of obesity. In addition, a WC cutoff value lower than the WHO standard should be implemented in Indonesia. In many Asian populations, a high prevalence of metabolic risk occurs at lower WC compared to that in Europeans. High awareness of “obesity is a disease” among the population, and health providers are indispensable as obesity prevalence and obesity-related chronic diseases are ramping up. Early diagnosis of obesity is needed to ensure early prevention and control of diabetes complications to decrease mortality and the economic and health burden of non-communicable diseases worldwide. Multicenter research with a larger population and a consensus held by experts are needed to determine on the optimal cutoff value of obesity and its association with the severity of comorbidities in Indonesia.

## Acknowledgments

The authors appreciate the kindly help of Dr Cicilia Marcella for her professional writing and English editing of the manuscript.

## Author contributions

All authors have seen and approved the manuscript and contributed significantly for the paper.

**Conceptualization:** Gaga Irawan Nugraha, Dicky L. Tahapary, Rachmad Wishnu Hidayat, Nurul Ratna M. Manikam, Mas Rizky A.A. Syamsunarno, Farid Kurniawan, Dwi Yuniati Daulay, Dante Saksono Harbuwono, Sidartawan Soegondo.

**Data curation:** Gaga Irawan Nugraha, Mas Rizky A.A. Syamsunarno, Farid Kurniawan, Dwi Yuniati Daulay, Sidartawan Soegondo.

**Formal analysis:** Mas Rizky A.A. Syamsunarno, Errawan R. Wiradisuria.

**Investigation:** Dicky L. Tahapary, Rachmad Wishnu Hidayat, Nurul Ratna M. Manikam, Mas Rizky A.A. Syamsunarno, Farid Kurniawan, Errawan R. Wiradisuria, Dante Saksono Harbuwono.

**Methodology:** Gaga Irawan Nugraha, Dicky L. Tahapary, Rachmad Wishnu Hidayat, Nurul Ratna M. Manikam, Farid Kurniawan, Errawan R. Wiradisuria, Dante Saksono Harbuwono, Sidartawan Soegondo.

**Supervision:** Gaga Irawan Nugraha, Dicky L. Tahapary, Rachmad Wishnu Hidayat, Nurul Ratna M. Manikam, Mas Rizky A.A. Syamsunarno, Farid Kurniawan, Errawan R. Wiradisuria, Dwi Yuniati Daulay, Dante Saksono Harbuwono.

**Visualization:** Sidartawan Soegondo.

**Writing – review & editing:** Gaga Irawan Nugraha, Dicky L. Tahapary, Rachmad Wishnu Hidayat, Nurul Ratna M. Manikam, Mas Rizky A.A. Syamsunarno, Farid Kurniawan, Errawan R. Wiradisuria, Dwi Yuniati Daulay, Dante Saksono Harbuwono, Sidartawan Soegondo.
